# Supplementary material for: Impact of direct-acting antiviral therapy for hepatitis C–related hepatocellular carcinoma
Source: PLoS One. 2020 May 22;15(5):e0233212. doi: 10.1371/journal.pone.0233212 (PMC7244104; doi:10.1371/journal.pone.0233212)
Supplement: Attachment — Submitted filename: Response to Reviewers.docx [file pone.0233212.s002.docx]

Response to the reviewers

**Reviewer #1:**

**1. Since the patients who treated with interferon (IFN)-based regimen were excluded in the enrollment per the study design, please correct the following sentence on page 11: “…30 patients received non-DAA therapy (did not receive DAA therapy?)…**”.

Answer 1: Thanks for your comment. We corrected this error. After refining the criteria for curative therapy (initial treatment with surgery or RFA), 24 patients received non-DAA therapy.

**2. Please make sure that HCV-related HCC BCLC stage 0-A patients who did not receive DAA therapy are 30 instead of 31 as that labeled in Figure 2B.**

Answer 2: Thanks for your comment. There were 31 patients (2 and 29 patients in stage 0 and stage A, respectively) in the non-DAA therapy group, and 30 of them received curative therapy in the initial analysis.

**3. Given the high HCC recurrence rate after curative therapy in this study compared with that reported in reference 6, the authors need to explain the possible mechanisms associated with HCC recurrence.**

Answer 3: The recurrence of HCC after DAA therapy was higher in this study at 50% (30/60) than in reference 6 (29.6%), since we included patients who showed complete HCC response to TACE therapy and repeated surgery or RFA. After excluding these patients, the recurrence rate in the DAA therapy group was 37.1% (13/35). The follow-up time was longer in this study than in reference 6 (20 months vs. 15 months), which might be another reason. We mentioned this in the discussion.

**4. Small case number especially patients in BCLC intermediate or advanced stages is the main shortcoming for this study.**

Answer 4: Thanks for your comment. Because of the small number of HCC patients in advanced stages, this study cannot provide solid evidence of the benefit of DAA in these stages. We mentioned this as the main shortcoming in the discussion.

**Reviewer #2:**

**1. In this study, the mean duration from HCC diagnosis to the initiation of DAA therapy was 8.9 months. Moreover, the mean duration from curative HCC therapy to DAA prescription was 11.5 months. It might cause an immortal time bias between patients with and those without DAA therapy after the diagnosis of HCC. There might be two methods to minimize this imbalanced bias. The first one is to perform the date of DAA initiation as a time-dependent covariate. For patients in the DAA group, they were classified as the DAA untreated group before the prescription of DAA (untreated period). They were enrolled as DAA treated group after they received DAA therapy (treated period, from DAA initiation to end of follow-up). The second one is to set a time window. All patients started to be followed up after the index date (maybe 9 months or 1 year after the date of HCC diagnosis). All events occurred before the index date were excluded from the analysis.**

Answer 1: Thanks for your precious comment. We chose the second method to exclude immortal time bias. After adjustment, the median periods of HCC diagnosis to DAA therapy and curative HCC therapy to DAA prescription were 25 and 24 months, respectively. We revised this in the methods, tables, and results.

**2. From the “data collection” in the section of “Materials and methods”, patients were enrolled from January 2010 to December 2019. Moreover, the last follow-up date was December 2019. It seemed that the longest follow-up period was 10 years (120 months). However, in the section of “Results”, the range of follow-up was 1-180 months. Moreover, in the Figure 2A, at least 7 patients (4 in the non-DAA group, and 3 in the DAA group, respectively) had a follow-up duration of 132 months or more. Please clarify it.**

Answer 2: Thanks for your comment. Eight patients (5 in the non-DAA group, and 3 in the DAA group) received HCC diagnosis before 2010 and received therapy during the study period of 2010–2019. The earliest date of diagnosis was December 2004. We mentioned this in the methods.

**3. In the Table 2, among the all 60 patients in the DAA group, 35 patients received HCC curative therapy. However, in the Page 11, Paragraph 2 (recurrent HCC after curative treatment), in the early stage (BCLC stage 0-A) of HCC patients receiving curative therapy, 48 patients received DAA therapy. Please to clarify it.**

Answer 3: Thanks for your comment. Thirteen patients who showed complete response to HCC after TACE or repeated therapy were included. We revised this in the results.

**4. In this study, TACE was regarded as a curative treatment. However, the overall survival rate and tumor control rate were lower in patients who underwent TACE when compared to resection surgery or local ablation therapy. It is suggested to classified TACE as the non-curative treatment modality. Moreover, in the Table 1, it is suggested to show the data of patient number according to their first treatment modality for HCC (for example, how many patients underwent resection surgery at the time of HCC diagnosis between DAA and non-DAA groups).**

Answer 4: Thanks for your comment. We excluded patients who received TACE therapy as a curative treatment and re-analyzed the data as suggested. We have provided the data on initial treatment modality in Table 1.

**5. It is suggested to provide the data of FIB-4 and albumin-bilirubin (ALBI) grade in the Table 1.**

Answer 5: Thanks for your comment. FIB-4 and albumin-bilirubin grade were vital tools to evaluate liver fibrosis and HCC prognosis. We added these data in the table.

**6. It is suggested to provide the median and IQR when presenting the data regarding the follow-up period**

Answer 6: Thanks for your comment. We revised all data on the follow-up periods by providing the median and IQR.
