# Supplementary material for: Impact of direct-acting antiviral therapy for hepatitis C–related hepatocellular carcinoma
Source: PLoS One. 2020 May 22;15(5):e0233212. doi: 10.1371/journal.pone.0233212 (PMC7244104; doi:10.1371/journal.pone.0233212)
Supplement: Attachment — Submitted filename: Response to reviewers.docx [file pone.0233212.s003.docx]

Response to the reviewers

**Reviewer #1:**

1. **The authors had revised my previous comments point-by-point in its 2nd version.**

Answer 1: Thanks for your comment.

**2. The term “non-DAA therapy” is easily interpreted as “any kinds of antiviral therapies other than DAA” by the readers, I suggest the authors adjust the statement appropriately and consistently in the context.**
Answer 2: Thanks for your comment. We have replaced the term “non-DAA therapy” to “no DAA group” and refined the definition of this group in the results section.

**3. Polish the language by a native English speaker and give the approval certificate to the editorial office.**

Answer 3: We have sent the manuscript to an English language editor and provided the associated certificate to the editorial office, as suggested.

**Reviewer #2: (No Response)**
